# Supplementary material for: Etiology of severe mastitis in French dairy herds
Source: PLoS One. 2023 Dec 14;18(12):e0295614. doi: 10.1371/journal.pone.0295614 (PMC10721090; doi:10.1371/journal.pone.0295614)
Supplement: S2 File — (PDF) [file pone.0295614.s002.pdf]

Lorem Ipsum

Lorem ipsum dolor sit amet, consectetur adipiscing elit. Mauris maximus fringilla ligula, in malesuada erat tempor ac. Quisque dapibus posuere turpis, vel aliquam massa vehicula non.

Veterinary clinics	A	B	C	D	E	F	G	H	I	J
E coli	20	33	7	15	48	32	39	31	31	117
other Gram negative	2	7		2	24		4	6	3	14
Staphylococcus aureus	4	6	4	3	14	4	4	11	2	16
Non *aureus Staphylococci* NAS)	3	2	1	1	12	1	2	4	1	5
Streptococcus uberis	3	3	3		16	5	6	16	3	21
Streptococcus dysgalactiae	1	2			2			1		6
autres strepto/entérocoques	3	4			10	2	1	7	1	1
Trueperella pyogenes					5			2	1	4
other Gram positive					1		2			
no growth	3	5			4	5	7	11	1	5
fungi or yeast										4
**Total**	**39**	**62**	**15**	**21**	**136**	**49**	**65**	**89**	**43**	**193**

Veterinary clinics	K	L	M	N	O	P	Q	R	S	Global values	Corrected values
E coli	205	210	57	143	15	10	20	67	18	1118	651
other Gram negative	14	11		10	3	2	9	6	11	128	90
Staphylococcus aureus	27	56	3	16	5		1	3	3	182	97
Non *aureus Staphylococci* NAS)	16	49	10	5	1	1	11	5	2	132	75
Streptococcus uberis	23	83	9	21	2	1	13	7	4	239	131
Streptococcus dysgalactiae	3	16		11	2			1	1	46	20
autres strepto/entérocoques	6	3		3	1	2	6	1	1	52	41
Trueperella pyogenes	2	1		13						28	15
other Gram positive				2						5	5
no growth	23	34	2	33			4	8	3	148	79
fungi or yeast		1		3					1	9	4
**Total**	**319**	**464**	**81**	**260**	**29**	**16**	**64**	**98**	**44**	2087	1210

S2 File 2 row data by vet clinics_202312012718-1.tif This is a preview of your figure rendered on a simulated PLOS journal page.

Maecenas ac est sit amet odio sollicitudin euismod. In risus odio, convallis a neque ac, varius ultricies arcu. Vestibulum et quam iaculis, ultricies odio et, molestie magna. Suspendisse vehicula purus id turpis eleifend, et convallis dui dignissim. Praesent tempus elit a metus sollicitudin, sed fringilla nulla porttitor. Nullam in tempus massa. Nunc maximus magna massa, nec volutpat risus rhoncus ut. Fusce quis ante sem. Aenean nulla nibh, tempus sit amet rhoncus at, eleifend vel risus. Sed dictum, sem ultrices elementum pharetra, lacus diam volutpat orci, scelerisque semper dui lacus ut enim.

Suspendisse in nunc id lacus commodo consequat. Proin semper aliquam varius. Fusce vitae neque aliquam nisi ultrices sodales vitae ut enim. Vivamus nec dictum ipsum. Sed condimentum ante eu urna tincidunt tincidunt. In ac lacus nec ipsum viverra volutpat posuere vel lacus. Class aptent taciti sociosqu ad litora torquent per conubia nostra, per inceptos himenaeos. Morbi rhoncus ipsum quis lorem hendrerit, at vulputate massa tempus. Ut arcu nisl, gravida vitae risus ultricies, porta venenatis massa. Cras dignissim, enim at faucibus aliquam, sapien nisl eleifend dolor, vel mollis nulla nisi id ipsum. Pellentesque vehicula ultricies risus sit amet faucibus. Praesent sit amet mi ac est faucibus accumsan. Praesent pulvinar sit amet orci auctor feugiat.

Phasellus vitae congue est. Duis rutrum iaculis nunc, sed sollicitudin neque eleifend nec. Pellentesque ac nisi eget tortor imperdiet sagittis ut in orci. Mauris porta convallis euismod. Donec in ultricies urna, nec interdum lectus. Nullam sit amet finibus augue, eget rutrum metus. Nam faucibus, urna ac finibus eleifend, neque nisi lobortis ante, at pharetra purus purus sed urna. Curabitur sit amet dui at enim porta posuere non vehicula ligula. Suspendisse potenti. Vestibulum arcu magna, vulputate a massa ac, molestie tincidunt dui.

Donec id tempus lacus, sed tristique nulla. Nullam rutrum risus ut pharetra porttitor. Nam mattis dolor erat, sed volutpat est mattis sed. Suspendisse eu porta tellus. Cras gravida velit sed maximus fermentum. Fusce vitae metus commodo, sagittis nunc sed, faucibus nunc. Integer iaculis quam mattis, luctus neque in, viverra magna. Nulla rhoncus feugiat orci, quis posuere ligula ornare at. Integer vel sagittis risus. Donec semper metus nec finibus accumsan. Mauris sit amet suscipit ante. Aliquam accumsan, nisl vitae vulputate elementum, turpis nibh varius urna, vel bibendum nulla nunc ac quam. Aenean malesuada egestas maximus. Pellentesque faucibus, odio at tincidunt ullamcorper, eros nisi pellentesque mi, non blandit sapien neque quis lectus.
