# Supplementary material for: Etiology of severe mastitis in French dairy herds
Source: PLoS One. 2023 Dec 14;18(12):e0295614. doi: 10.1371/journal.pone.0295614 (PMC10721090; doi:10.1371/journal.pone.0295614)
Supplement: S3 File — (PDF) [file pone.0295614.s003.pdf]

Lorem Ipsum

Lorem ipsum dolor sit amet, consectetur adipiscing elit. Mauris maximus fringilla ligula, in malesuada erat tempor ac. Quisque dapibus posuere turpis, vel aliquam massa vehicula non.

Annex 3

Comparison between results obtained from veterinary clinic milk culture and obtained by MALDI TOF

377 bacterial strains, isolated from mastitis milk (clinical or subclinical) and identified by the milk culture method described in this article, were sent to the LABOCEA – Fougères laboratory for bacteriological analysis with a MALDITOF.

Gram-		MALDI TOF								
		EC	*Klebsiella*	*Serratia*	Pseudo.	Entero.	Citro.	*Proteus*	*Hafnia*	Total
Milk cult. Vet. Clinic	EC	40							1	41
	Klebsiella		11	4		4	3			22
	Serratia		1	8		1	2			12
	Pseudo.			2	2			1		5
	Gram-			7			2	1		10
	Total	40	12	21	2	5	7	2	1	90

Table 1 : correspondence between bacteriological test results obtained with the method described and those obtained with MALDITOF : Gram- bacteria

(EC = *Escherichia coli*, Pseudo. = *Pseudomonas*, Entero. = Enterobacter, Citro. = Citrobacter)

Gram+		MALDI TOF							
		SA	NaS	SU	SD	*Enterococcus*	TP	Other G+	Total
Milk cult. Vet. Clinic	SA	49	2					1	52
	NaS	1	35	1				3	40
	SU			124	2			12	138
	SD				19			1	20
	Enterococcus		1			14		1	16
	TP						16	1	17
	Other G+		1	1	1			1	4
	Total	50	39	126	22	14	16	20	287

Table 2 : correspondence between bacteriological test results obtained with the method described and those obtained with MALDITOF : Gram+ bacteria

(SA = *Staphylococcus aureus*, NaS = non *aureus Staphylococcus*, SU = *Streptococcus uberis*, SD = *Streptococcus dysgalactiae*, TP = *Trueperella pyogenes*)

S3 File 3 comparison vet milk culture with maldi Tof_202312010704-1.tif This is a preview of your figure rendered on a simulated PLOS journal page.

Maecenas ac est sit amet odio sollicitudin euismod. In risus odio, convallis a neque ac, varius ultricies arcu. Vestibulum et quam iaculis, ultricies odio et, molestie magna. Suspendisse vehicula purus id turpis eleifend, et convallis dui dignissim. Praesent tempus elit a metus sollicitudin, sed fringilla nulla porttitor. Nullam in tempus massa. Nunc maximus magna massa, nec volutpat risus rhoncus ut. Fusce quis ante sem. Aenean nulla nibh, tempus sit amet rhoncus at, eleifend vel risus. Sed dictum, sem ultrices elementum pharetra, lacus diam volutpat orci, scelerisque semper dui lacus ut enim.

Suspendisse in nunc id lacus commodo consequat. Proin semper aliquam varius. Fusce vitae neque aliquam nisi ultrices sodales vitae ut enim. Vivamus nec dictum ipsum. Sed condimentum ante eu urna tincidunt tincidunt. In ac lacus nec ipsum viverra volutpat posuere vel lacus. Class aptent taciti sociosqu ad litora torquent per conubia nostra, per inceptos himenaeos. Morbi rhoncus ipsum quis lorem hendrerit, at vulputate massa tempus. Ut arcu nisl, gravida vitae risus ultricies, porta venenatis massa. Cras dignissim, enim at faucibus aliquam, sapien nisl eleifend dolor, vel mollis nulla nisi id ipsum. Pellentesque vehicula ultricies risus sit amet faucibus. Praesent sit amet mi ac est faucibus accumsan. Praesent pulvinar sit amet orci auctor feugiat.

Phasellus vitae congue est. Duis rutrum iaculis nunc, sed sollicitudin neque eleifend nec. Pellentesque ac nisi eget tortor imperdiet sagittis ut in orci. Mauris porta convallis euismod. Donec in ultricies urna, nec interdum lectus. Nullam sit amet finibus augue, eget rutrum metus. Nam faucibus, urna ac finibus eleifend, neque nisi lobortis ante, at pharetra purus purus sed urna. Curabitur sit amet dui at enim porta posuere non vehicula ligula. Suspendisse potenti. Vestibulum arcu magna, vulputate a massa ac, molestie tincidunt dui.

Donec id tempus lacus, sed tristique nulla. Nullam rutrum risus ut pharetra porttitor. Nam mattis dolor erat, sed volutpat est mattis sed. Suspendisse eu porta tellus. Cras gravida velit sed maximus fermentum. Fusce vitae metus commodo, sagittis nunc sed, faucibus nunc. Integer iaculis quam mattis, luctus neque in, viverra magna. Nulla rhoncus feugiat orci, quis posuere ligula ornare at. Integer vel sagittis risus. Donec semper metus nec finibus accumsan. Mauris sit amet suscipit ante. Aliquam accumsan, nisl vitae vulputate elementum, turpis nibh varius urna, vel bibendum nulla nunc ac quam. Aenean malesuada egestas maximus. Pellentesque faucibus, odio at tincidunt ullamcorper, eros nisi pellentesque mi, non blandit sapien neque quis lectus.
