# Supplementary material for: Etiology of severe mastitis in French dairy herds
Source: PLoS One. 2023 Dec 14;18(12):e0295614. doi: 10.1371/journal.pone.0295614 (PMC10721090; doi:10.1371/journal.pone.0295614)
Supplement: Attachment — Submitted filename: Response to Reviewers.docx [file pone.0295614.s004.docx]

Response to Reviewers

Dear reviewers, thank you very much for reviewing this article. I hope you find my changes and additions satisfactory.

Reviewer #1

Inconsistent use of abbreviations. For example, in the case of E. coli, between Escherichia coli, E. coli, and EC. It is best to write out and abbreviate once the first time it is mentioned, and then use the abbreviation consistently. This is also the case for other bacteria. rectified
L 17: Is severe mastitis really ”frequent” ? Reword please, I agree, sorry, it was a mistake, rectified
L29: Please explain. What is the difference in severe mastitis treatment if you know the pathogen several hours later completed
L 54: Please find another word for: short-term lethality rectified
L 59: Delete “ and so forth” rectified
Ll65-67: Quinolones are also used in other areas of the world. Reword please rectified
L 68: CM instead of MC rectified; Why you should investigate the pathogen if you treat every severe case with quinolones? There are two reasons to do a bacteriological exam : first, French rules enforced it because for using fluoroquinolones you must get an bacteriological exam result and an antibiotic sensitivity test and second, fluoroquinolones are very effective against the most prevalent mammary pathogens isolated from severe mastitis except Streptococcus. So, bacteriological exam allows to rectify antibiotic treatment when Streptococcus is isolated, completed.
L 83: Did you do somatic cell count testing? If not, delete “cyto-“ rectified
L 85: 30 microliters in total or per single plate? 30 microliters per single plate, rectified
L105-106: Is the sample considered contaminated from 1 colony of three different species or from 5 colonies of at least 3 different species? Sample is considered contaminated from 1 CFU of at least 3 different species, completed
L 108: a dot is missing after spp rectified
L 109: It would be good if you not only describe your method, but also critically evaluate it (possibly this will come in the discussion). It is evaluated and completed (Annex 3) in the Discussion
L130: Table 2 is missing. Or is table 3 actually table 2? Sorry, it was a mistake, there is no table 3, just tables 1 and 2, rectified
L132: Does str. uberis account for 8-20% of the results? Table 3 also mentions 7% in some cases. See Farm I or R Sorry, another mistake, *Strep. uberis* account for 7-20% of the results, rectified
L 139: The sentence is very colloquial, please reword the sentence. rectified
L 151: catalase negative rectified
L175ff/ Table 1: Inconsistent writing: Once comma (,) and once dots (.) are used for the percentages. In addition, when rounding to one position behind the decimal point, it is better to omit the second 0 or to remain consistent. Sorry, rectified
L 179: please explain 60 microliters or 30? 30 microliters per plate for 2 plates where each colony (Gram+ [COS and CAN] or Enterobacteriaceae [COS and HEKT]) can grow, so a total of 60 microliters
L 187: Please explain, when you mention it for the 1st time “leading French dairy regions”. What are the leading dairy regions in France? Brittany, Normandy, Burgundy/Franche Comté, Grand Est region, Auvergne, completed

References: Sometimes with the link from the internet and sometimes without it completed

Table 2: Is there also a table 2? Just table 1 and table 2, no table 3, sorry

Reviewer #2: In this report, Salat and colleagues report on the prevalence of the major pathogens isolated from severe clinical mastitis cases in France.
Data are clearly reported. Manuscript is well written with only a few corrections needed.

I have the following comments:
- L78: delay between call and sampling? please provide details of the sampling procedure completed
- L80: provide clinical report form as supplementary completed (Annex 2)
- L104: specificity of the detection method? reference? evaluated and completed in Discussion and Annex 3
- Were samples resulting in more than 3 colony types considered contaminated samples? Yes, this was completed
- L110-117: this paragraph should be placed at the beginning of Methods section. rectified
- How was Trueperella pyogenes identified? Completed Could the authors provide a decision diagram? diagrams 1 and 2 added
- L130: please revise table number: table 2 is indicated in the text while the title of the table indicates Table 3. Sorry, this was a mistake, rectified
- L131-132: please provide the number of clinics for which S. uberis was the second most frequent pathogen (11/19). Authors could also state that S. aureus is the third pathogen in prevalence (6/19). rectified
- L145: could the authors be more precise? Are these their own data? I think these data should be presented. Yes these are our own data, and they are presented in Annex 3
- L149: period at end of sentence missing. Rectified
